# Disability level’s impact on blood pressure-mortality association in older long-term care adults: evidence from a large Chinese cohort study

**DOI:** 10.1186/s12877-024-05094-y

**Published:** 2024-05-31

**Authors:** Yue Zhong, Chuanteng Feng, Lisha Hou, Ming Yang, Xinjun Zhang, Jinhui Wu, Birong Dong, Peng Jia, Shujuan Yang, Qingyu Dou

**Affiliations:** 1 Department of Cardiology, West China Hospital, Sichuan University, Chengdu, China; 2 Institute for Disaster Management and Reconstruction, Sichuan University-The Hongkong Polytechnic University, Chengdu, Sichuan China; 3 West China School of Public Health and West China Fourth Hospital, Sichuan University, Chengdu, China; 4 National Clinical Research Center for Geriatrics, Center of Gerontology and Geriatrics, West China Hospital, Sichuan University, Chengdu, China; 5 School of Resource and Environmental Sciences, Wuhan University, Wuhan, China; 6 International Institute of Spatial Lifecourse Health (ISLE), Wuhan University, Wuhan, China; 7 West China Hospital of Sichuan University, 37 Guoxue Alley, Wuhou District, Chengdu, 610041 Sichuan China

**Keywords:** Older adults, Disability, Blood pressure, Long-term care

## Abstract

**Background:**

Evidence of the optimal blood pressure (BP) target for older adults with disability in long-term care is limited. We aim to analyze the associations of BP with mortality in older adults in long-term care setting with different levels of disability.

**Methods:**

This prospective cohort study was based on the government-led long-term care programme in Chengdu, China, including 41,004 consecutive disabled adults aged ≥ 60 years. BP was measured during the baseline survey by trained medical personnel using electronic sphygmomanometers. Disability profile was assessed using the Barthel index. The association between blood pressure and mortality was analyzed with doubly robust estimation, which combined exposure model by inverse probability weighting and outcome model fitted with Cox regression. The non-linearity was examined by restricted cubic spline. The primary endpoint was all-cause mortality, and the secondary endpoints were cardiovascular and non-cardiovascular mortality.

**Results:**

The associations between systolic blood pressure (SBP) and all-cause mortality were close to a U-shaped curve in mild-moderate disability group (Barthel index ≥ 40), and a reversed J-shaped in severe disability group (Barthel index < 40). In mild-moderate disability group, SBP < 135 mmHg was associated with elevated all-cause mortality risks (HR 1.21, 95% CI, 1.10–1.33), compared to SBP between 135 and 150 mmHg. In severe disability group, SBP < 150 mmHg increased all-cause mortality risks (HR 1.21, 95% CI, 1.16–1.27), compared to SBP between 150 and 170 mmHg. The associations were robust in subgroup analyses in terms of age, gender, cardiovascular comorbidity and antihypertensive treatment. Diastolic blood pressure (DBP) < 67 mmHg (HR 1.29, 95% CI, 1.18–1.42) in mild-moderate disability group and < 79 mmHg (HR 1.15, 95% CI, 1.11–1.20) in severe disability group both demonstrated an increased all-cause mortality risk.

**Conclusion:**

The optimal SBP range was found to be higher in older individuals in long-term care with severe disability (150-170mmHg) compared to those with mild to moderate disability (135-150mmHg). This study provides new evidence that antihypertensive treatment should be administered cautiously in severe disability group in long-term care setting. Additionally, assessment of disability using the Barthel index can serve as a valuable tool in customizing the optimal BP management strategy.

**Trial registration:**

Chinese Clinical Trial Registry (Registration Number: ChiCTR2100049973).

**Supplementary Information:**

The online version contains supplementary material available at 10.1186/s12877-024-05094-y.

## Background

Hypertension stands as one of the most widespread diseases among the older adults, affecting approximately 60% of individuals aged over 60 [1, 2]. With the pace of population ageing accelerating [3], more than half of older individuals grapple with disabilities in activities of daily living (ADL), which is a significant health issue among older adults [4, 5]. Unfortunately, this particular demographic is often excluded from randomized controlled trials (RCT) focused on antihypertensive treatment for older adults [6–8]. Studies like SPRINT (Systolic Blood Pressure Intervention Trial) and the recent STEP (Strategy of Blood Pressure Intervention in the Elderly Hypertensive Patients) trial have demonstrated the benefits of intensive blood pressure (BP) control for older patients [9, 10]. But these data only represent as few as one third of older adults in the general population, since they excluded patients with multimorbidity or functional loss [7, 11], raising the need for research specifically focus on this unique group.

Current guidelines recommend individualized treatment approaches for older individuals with impaired function, but the detailed BP target for this group is lack of evidence [12–15]. On one hand, some observational cohort studies focusing on the oldest individuals have revealed that both lower and higher systolic BP (SBP) levels (< 120 mmHg and > 165 mmHg) are associated with increased mortality risk, presenting a J- or U-shaped association [16–18]. On the other hand, there have been suggestions that elevated SBP (> 140 mmHg) does not lead to excess mortality. In fact, it may even be inversely correlated with an increased risk of death in older individuals with poor functioning [19, 20]. These conflicting findings may stem from the varying degrees of disability in the above studies, which potentially change the association between BP and mortality.

Given the ethical constraints associated with conducting RCTs in older individuals with functional disabilities, real-world data emerges as a crucial resource to bridge the evidence gap for personalized treatment. This current study draws upon the government-backed Chengdu Long-Term Care Insurance (LTCI) cohort, encompassing older individuals with varying degrees of ADL disability who applied for LTCI since 2017 [21]. Our objectives were to investigate the non-linear association between BP levels and mortality in older adults with ADL disability in government-backed long-term care setting, and to evaluate how different profiles of disability could change the association between BP and the risks of all-cause mortality as well as cause-specific mortality.

## Methods

### Study design

This is a prospective open cohort study. The participants were enrolled from the government-led LTCI programme in Chengdu, initiated on September 27th, 2017, and overseen by the Chengdu Healthcare Security Administration [21]. The inclusion criteria were: (1) older adults requiring long-term care, with sustained loss of ADL independence persisting for over 6 months, irreversibly unresponsive to rehabilitation, and (2) possess urban medical insurance [21]. The Chinese government promotes a long-term care system with home-based care as its cornerstone, complemented by community-based services and institutional care. Under the support of LTCI, care is provided in two manners: (1) home care by a family member with home visits from a nursing home supporter, and (2) institutional care in a nursing home. Initially, a total of 44,258 participants were enrolled. Exclusions comprised individuals below the age of 60 and those with functional disabilities resulting from trauma. Each participant was tracked from their entry date to either the date of decease or August 2nd, 2021, whichever came first. Ultimately, a cohort of 41,004 individuals aged 60 and above were recruited between September 27th, 2017, and August 2nd, 2021 (Supplemental Fig. S1). The study received ethical approval from the institutional ethics review committee of West China Hospital (2017 − 303) and was registered at the Chinese Clinical Trial Registry (Registration Number: ChiCTR2100049973, Date: 15/08/2021). The study adhered to the Helsinki Declaration of 1964. Written informed consent was obtained from all participants or their legal representatives.

### Outcomes

The primary outcome was all-cause mortality. Data regarding deaths were sourced from the national medical insurance systems, which were linked to the electronic medical records and corresponding death certificates. Secondary outcomes were cardiovascular and non-cardiovascular mortalities, which were classified in accordance with the International Classification of Diseases, 10th revision (ICD-10 codes: I10-I15, I20-I25, I30-I52, I60-I79, and I95-I99, representing cardiovascular mortality).

### Measurement of blood pressure

The primary predictor variable was BP. At the time of applying for LTCI, the government will send trained medical workers to assess the functional status of the applicants. The vital signs including BP was assessed during this baseline survey. BP was measured by Omron electronic sphygmomanometer (HEM7122 and HEM7124). These measurements followed the standardized BP measurement protocol outlined in established guidelines [12, 14, 15]. Prior to use, the electronic sphygmomanometers were calibrated, and all investigators underwent uniform training. For each participant, three accurate BP readings were taken on the right arm in a rested state. These readings were then averaged to calculate both the SBP and diastolic BP (DBP). In cases where participants were bedbound, BP measurements were obtained in a recumbent position.

### Measurement of disability profiles

A structured face-to-face interview was conducted by trained investigators, during which information was gathered from participants or their caregivers. The entire evaluation process was recorded on video, and a committee overseeing the LTCI programme was entrusted with the assessment. The participants’ disability profiles were assessed by the Barthel index, a widely recognized instrument for measuring performance in basic ADL [22]. This index encompasses 10 essential daily activities, including feeding, bathing, grooming, dressing, bowel and bladder control, toilet use, steps, transfer and mobility. Scores range from 0 to 100 points. According to the current policy of China’s LTIC programme, severe disability was determined as the Barthel index < 40), while mild to moderate disability was the index ≥ 40 [21]. This criterion is consistent with the standardized disability assessment criteria used across the 15 LTCI cities in China [21]. Validation study indicated with a Barthel score < 40, no one was independent in the mobility skills and fewer than 50% were independent in the very basic skills [23].

### Covariates

Covariates were meticulously gathered through a standardized face-to-face questionnaire and physical examination, and the diagnosis of chronic disease was confirmed by related medical records. When the disabled older adults applied for LTCI, they were required to provide official medical records detailing their medical history and condition as proof. Demographic characteristics included age, gender, education level, and marital status. Participants’ care mode was classified as either home-based or institutional. Comorbidities included coronary artery disease, heart failure, chronic obstructive pulmonary disease, cerebrovascular disease, diabetes, chronic kidney disease at stages 3 to 5, and cancers. The comorbidity score was assessed using the Charlson comorbidity index [24]. The diagnosis of hypertension is confirmed by diagnosis certificates from medical institutions or current use of antihypertensive medication. Information regarding antihypertensive drug treatment at baseline was collected from the latest medical records, as well as the laboratory indicators including total cholesterol, fasting plasma glucose, serum creatinine, and blood uric acid. The cognitive impairment and perception impairment were evaluated by the cognitive and sensory perception assessment scale, as previously reported [25]. The scale demonstrated a credible level of internal consistency, with a Cronbach’s alpha coefficient of 0.91. The cognitive impairment assessment focused on memory and concentration abilities, while perception impairment evaluation encompassed vision, hearing, and communication capacities.

### Statistical analyses

Continuous variables were presented as mean and standard deviation, while categorical variables as numbers and percentages. Baseline data between the two disability groups were compared using t-tests or χ² tests. The measure of person-years is the cumulative survival time of all the participants during our follow-up. Inverse propensity weighting was used to balance the distribution of baseline covariates in subsequent analyses. The study employed a doubly robust approach to evaluate the non-linear association between the BP levels and mortality risk. This entailed employing an exposure model through inverse probability weighting, and an outcome model fitted with a Cox regression model and restricted cubic splines [26, 27]. Both SBP and DBP were treated as continuous variables to assess their non-linear association with all-cause mortality, as well as cardiovascular and non-cardiovascular mortality. Reference points were defined as 150 mmHg for SBP and 90 mmHg for DBP, based on significant benefits observed in prior RCT upon lowering SBP to < 150 mmHg in older adults [6]. Restricted cubic splines were employed to graphically estimate potential non-linear associations between BP and mortality in the weighted samples. For the non-linear trend, we defined a ‘U-shaped’ curve as a basically symmetrical shape across the nadir with increasing mortality risk at both higher and lower levels of BP, while a ‘reversed J-curve’ was characterized by an asymmetrical shape with an augmentation of mortality risk with decreasing BP from the nadir [28]. The cut-off points were determined by the intersections of the 95% confidence intervals (CI) of mortality risk with the abscissa where hazard ratio (HR) was 1. Subsequently, BP values were categorized into ranges based on the identified cut-off points. Cox proportional hazards models, adjusted for the aforementioned covariates, were utilized to estimate the association between BP ranges and mortality. Fine-Gray competing models were employed to assess the association between categorical SBP and DBP with cause-specific mortality, in consideration of potential competing risks [29].

Subgroup analyses were conducted among: (1) participants aged 60 to 80 years and those over 80 years; (2) males and females; (3) individuals with and without antihypertensive treatment; (4) individuals with and without cardiovascular comorbidity; and (5) individuals with comorbidity scores exceeding and not exceeding the median level. Categorical BP ranges derived from restricted cubic splines were assessed in all models. Furthermore, Cox regression with interaction terms was used to test for significance of differences between subgroups. Sensitivity analyses including: (1) stratification of participants based on every 10-mmHg increment of the BP value; (2) exclusion of participants with a follow-up period < 6 months to preclude excessively low BP values influenced by terminal phases; (3) exclusion of participants with cancer, given their typically abbreviated survival periods; and (4) fitting of Cox proportional-hazards regression models without doubly robust estimators.

The threshold for statistical significance was set at a two-sided *p* < 0.05 for all analyses. The statistical software R (Version 3.4.3; R Foundation for Statistical Computing, Vienna, Austria) was employed for all analyses.

## Results

### Participant characteristics

At baseline, the mean age of the 41,004 participants was 80.8 ± 9.1 years, with females comprising 57.1% (23,415) of the cohort. Table 1 provides a comprehensive description of participant characteristics. 71% of the participants (29,133) received home-based care. Notably, the mild-moderate disability group exhibited significantly higher levels of both SBP and DBP compared to those with severe disability. Over a median follow-up period of 14.2 months, a total of 17,797 participants (43.4%) died. Among these, 5,739 individuals died from cardiovascular causes, while 12,058 deaths were attributed to non-cardiovascular causes. The primary reasons for non-cardiovascular mortality were respiratory failure, infectious diseases and malnutrition related diseases.

**Table 1:** Baseline characteristics of the participants from the long-term care program in Chengdu grouped by disability status

Characteristics	All participants (*n* = 41,004)	Mild-moderate disability (*n* = 8,135)	Severe disability (*n* = 32,869)	*p* value**
Demographics				
Age (year)	80.75 ± 9.06	79.34 ± 9.23)	81.09 ± 8.99	**< 0.001**
Female sex	23,415 (57.1)	4,376 (53.8)	19,039 (57.9)	**< 0.001**
Educational level				**0.002**
Primary school or below	26,270 (64.1)	5330 (65.5)	20,940 (63.7)	
Middle school or above	14,734 (35.9)	2805 (34.5)	11,929 (36.3)	
Marriage status				**< 0.001**
In marriage	22,060 (53.8)	4,730 (58.1)	17,330 (52.7)	
Never married, widowed, or divorced	18,944 (46.2)	3,405 (41.9)	15,539 (47.3)	
**Care mode**				**< 0.001**
Home based	29,133 (71.0)	6,593 (81.0)	22,540 (68.6)	
Institutional based	11,871 (29.0)	1,542 (19.0)	10,329 (31.4)	
**Antihypertensive drugs use**				**0.001**
Yes	24,099 (58.8)	4,908 (60.3)	19,191 (58.4)	
No	16,905 (41.2)	3227 (39.7)	13,678 (41.6)	
Total cholesterol (mmol/L)*	5.22 ± 2.54	5.16 ± 2.17	5.23 ± 2.64	0.096
Fasting plasma glucose (mmol/L)*	6.23 ± 3.82	6.33 ± 3.78	6.20 ± 3.83	0.058
Serum creatinine (µmol/L)*	92.31 ± 46.71	92.94 ± 49.69	92.12 ± 45.81	0.332
Blood uric acid*	384.68 ± 166.04	386.62 ± 155.21	384.11 ± 169.06	0.358
**Chronic conditions**				
Coronary artery disease	10,199 (24.9)	1,941 (23.9)	8,258 (25.1)	**0.019**
Heart failure	4,567 (11.1)	859 (10.6)	3,708 (11.3)	0.067
Chronic obstructive pulmonary disease	12,609 (30.8)	2,451 (30.1)	10,158 (30.9)	0.179
Cerebrovascular disease	20,857 (50.9)	3,851 (47.3)	17,006 (51.7)	**< 0.001**
Diabetes mellitus	10,858 (26.5)	2,225 (27.4)	8,633 (26.3)	**0.048**
Chronic kidney disease stages 3–5	4,783 (11.7)	1,019 (12.5)	3,764 (11.5)	**0.007**
Cancer	2,156 (5.3)	414 (5.1)	1,742 (5.3)	0.463
**Cognitive impairment**				**< 0.001**
Robust	1,588 (3.9)	689 (8.5)	899 (2.7)	
Mild impairment	12,304 (30.0)	3,414 (42.0)	8,890 (27.0)	
Moderate impairment	19,248 (46.9)	3,264 (40.1)	15,984 (48.6)	
Severe impairment	7,864 (19.2)	768 (9.4)	7,096 (21.6)	
**Perception impairment**				**<0.001**
Robust	4,421 (10.8)	2,130 (26.2)	2,291 (7.0)	
Mild impairment	20,602 (50.2)	4,937 (60.7)	15,665 (47.7)	
Moderate impairment	14,388 (35.1)	1,043 (12.8)	13,345 (40.6)	
Severe impairment	1,593 (3.9)	25 (0.3)	1,568 (4.8)	
**Systolic blood pressure (mmHg)**	137.04 ± 25.24	143.14 ± 25.22	135.53 ± 25.01	**< 0.001**
**Diastolic blood pressure (mmHg)**	76.58 ± 15.30	79.15 ± 14.76	75.94 ± 15.37	**< 0.001**
**Charlson comorbidity index**	2.15 ± 1.74	2.11 ± 1.73	2.16 ± 1.74	**0.007**

Data was expressed as mean ± standard deviation for continuous variables, and as number (%) for categorical variables

*Data missing in this line, *n* = 4,321 for mild-moderate disability group, *n* = 14,876 for severe disability group

**Significant *p* values were shown in bold. The two disability groups were compared by t-tests or χ² tests as appropriate

### Association of BP with all-cause mortality risk

**Fig. 1:**
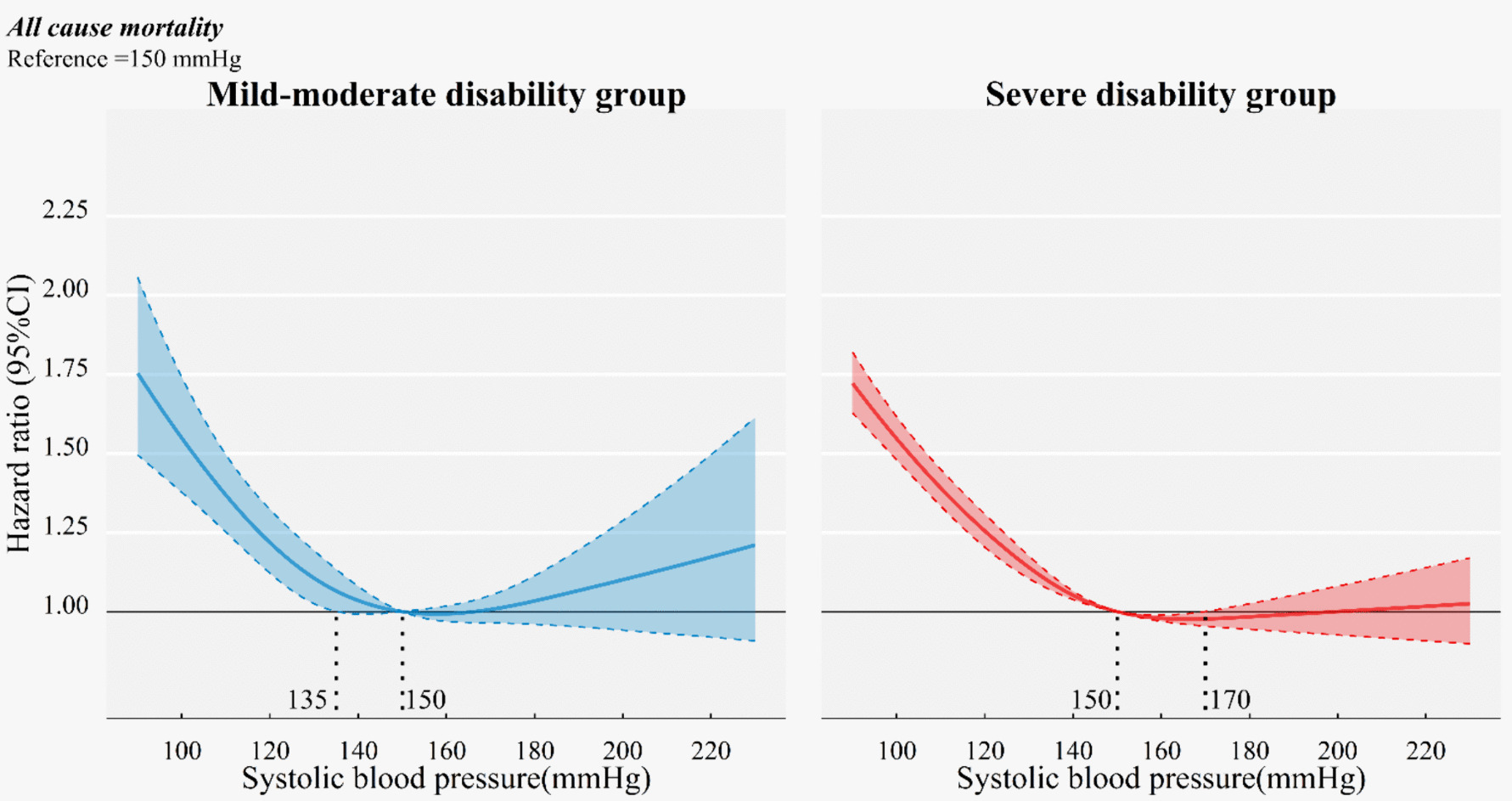
Cox models with cubic restricted splines of the association between systolic blood pressure and all-cause mortality risk stratified by disability status (reference: 150 mmHg). U-shaped association was observed in mild-moderate disability group, and reversed J-shaped in severe disability group. Hazard ratio was adjusted for age, sex, education, marital status, care modes, multimorbidity, cognitive impairment and perception impairment

The mild-moderate disability group exhibited a mortality rate of 18.7 per 100 person-years, while the severe disability group had a higher rate of 32.3 per 100 person-years. Among the mild-moderate disability group, we observed a nearly U-shaped curve (*p* < 0.001 for non-linear association) in the association between SBP and all-cause mortality. While a reversed J-curve was found among the severe disability group (*p* < 0.001 for non-linear association) (Fig. 1).

For the mild-moderate disability group, with 150 mmHg selected as the reference point, 135 mmHg was identified as the intersection of the 95% CI with the abscissa. Those with SBP < 135 mmHg exhibited a significantly increased risk of mortality, with the HR escalating from 1.06 (95% CI 1.01–1.13) at 135 mmHg to 1.54 (95% CI, 1.38–1.74) at 100 mmHg. In the severe disability group, 170 mmHg marked the intersection of the 95% CI with the abscissa. When compared to 150 mmHg, SBP of 150–170 mmHg showed a slightly lower risk of mortality [ HR (95% CI) at 160 mmHg: 0.98 (0.97–0.99)] (Fig. 1 and Supplemental Fig. S2). The association of DBP with all-cause mortality is depicted in Supplemental Fig. S3, where a reversed J-shaped association was observed in both the mild-moderate disability and severe disability groups.

The comparisons of the optimal BP ranges are detailed in Table 2. The middle ranges of BP derived from the cubic spline were the reference points. Compared with SBP of 135–150 mmHg, the mild-moderate disability group with SBP < 135 mmHg demonstrated an increased adjusted mortality risk (HR 1.21, 95% CI, 1.10–1.33). In severe disability group, SBP < 150 mmHg was associated with an elevated mortality risk (HR 1.21, 95% CI, 1.16–1.27) compared to SBP of 150–170 mmHg. Furthermore, DBP < 67 mmHg (HR 1.29, 95% CI, 1.18–1.42) in the mild-moderate disability group and < 79 mmHg (HR 1.15, 95% CI, 1.11–1.20) in severe disability group demonstrated an increased all-cause mortality risk. When BP values were divided by 10 mmHg, similar trends were observed as in the main analysis (Supplemental Table S1).

**Table 2:**
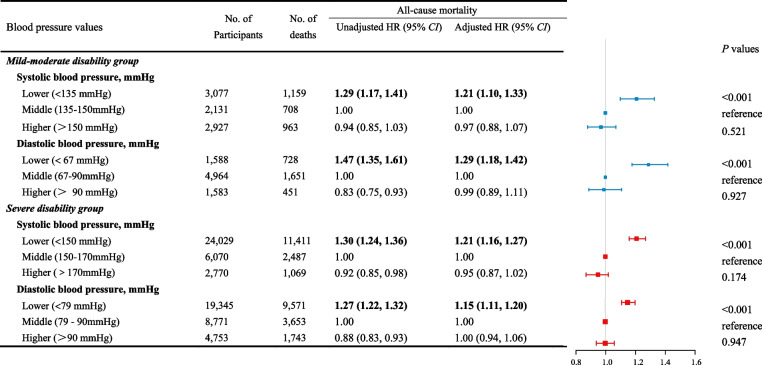
Association of blood pressure range with all-cause mortality risk stratified by disability status

HR values were shown in bold if significant difference

### Association of BP with cause-specific mortality risk

**Fig. 2:**
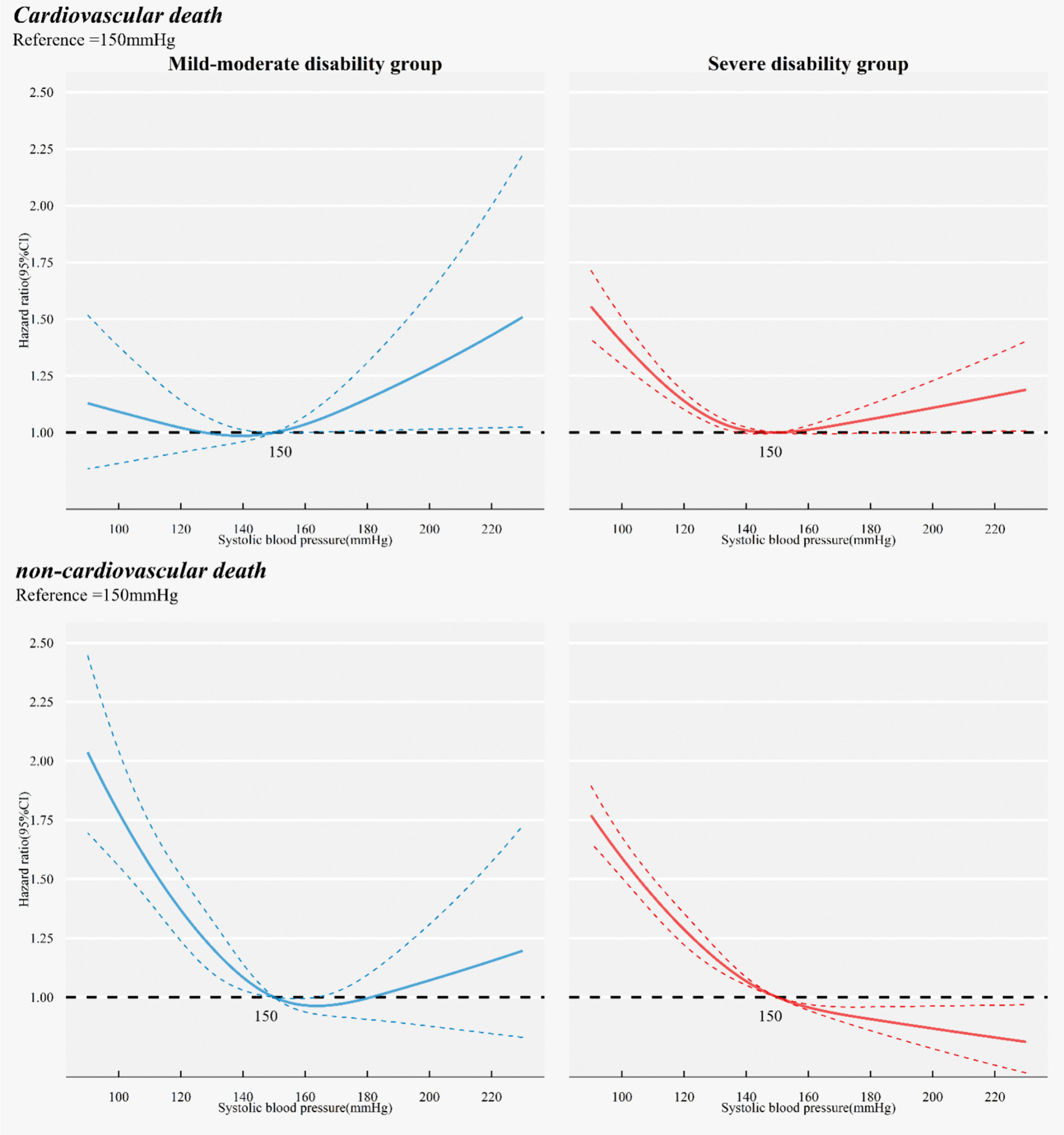
Cox model with cubic restricted splines analysis of systolic blood pressure with cardiovascular and non-cardiovascular mortality risk stratified by disability status (reference: 150 mmHg). Hazard ratio was adjusted for age, sex, education, marital status, care modes, multimorbidity, cognitive impairment and perception impairment

For cardiovascular mortality, in the mild-moderate disability group, SBP displayed a J-shaped association, whereas in the severe disability group, a reversed J-shaped association was observed (Fig. 2 ). Specifically, compared to those with SBP of 150–170 mmHg, individuals in the severe disability group with SBP < 150 mmHg exhibited a significantly higher cardiovascular mortality risk (HR 1.14, 95% CI, 1.06–1.24). Conversely, in the mild-moderate disability group, SBP < 135 mmHg conveyed no significant cardiovascular mortality risk (Fig. 2 and Supplemental Table S2). Additionally, when BP was categorized in 10 mmHg increments, lower SBP levels in the severe disability group were still significantly associated with increased cardiovascular mortality risk (Supplemental Table S3).

The association between SBP and non-cardiovascular mortality risk presented a reversed J-shaped curve in the mild-moderate disability group. While in the severe disability group, there was a negative correlation with increasing SBP (Fig. 2). Specifically, mild-moderate disability participants with SBP < 135 mmHg showed an increased non-cardiovascular mortality risk (HR 1.31, 95% CI, 1.17–1.47) compared to SBP of 135–150 mmHg (Supplemental Table S2). Furthermore, DBP < 79 mmHg in the severe disability group (HR 1.17, 95% CI, 1.11–1.23), as well as DBP < 67 mmHg in the mild-moderate disability group (HR 1.41, 95% CI, 1.27–1.57), were associated with an increased non-cardiovascular mortality risk. When BP was categorized in 10 mmHg increments, lower SBP levels were associated with increased non-cardiovascular mortality risk in both groups (Supplemental Table S4).

### Subgroup and sensitivity analyses

**Fig. 3:**
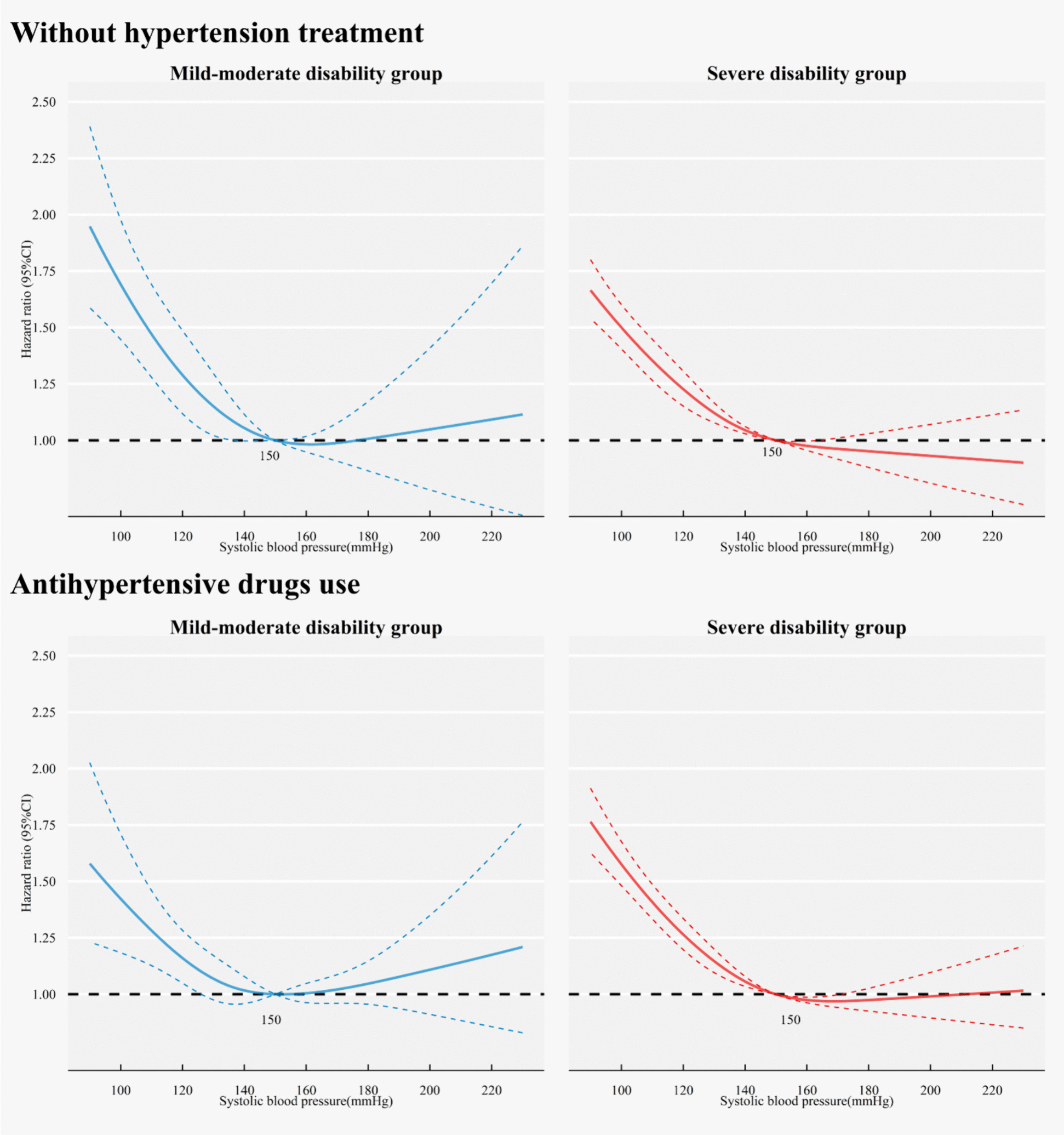
The association between SBP and all-cause mortality in participants with and without antihypertensive drug use

The subgroup analyses corroborated the main findings. No significant interactions were identified between SBP and all-cause mortality by subgroups defined by age, gender, antihypertensive treatment, cardiovascular morbidity, or the severity of comorbidity (all interaction *p* > 0.05, Supplemental Fig. S4). Notably, the use of antihypertensive drug did not alter the observed association between SBP and mortality (Fig. 3). Both SBP < 135 mmHg in the mild-moderate disability group and SBP < 150 mmHg in the severe disability group were associated with increased all-cause mortality risk across the subgroups (Supplemental Fig. S4). Additionally, the inflection points of mortality risk were generally lower than 150 mmHg in the mild-moderate disability subgroups and higher than 150 mmHg in the severe disability subgroups, indicating that the safe BP range for the severe disability group was consistently higher than that for the mild-moderate group (Supplemental Fig. S5).

When the model without doubly robust estimators was employed, the severe disability group exhibited a more pronounced decrease in the reverse J-shaped curve depicting the association between SBP and all-cause mortality. Minor variations were observed when participants with a follow-up period < 6 months and those with cancer were excluded in sensitivity analyses (Supplemental Fig. S6).

## Discussion

In this large open cohort study, we observed that disability status modified the association between SBP and mortality risk in long-term care settings. Older individuals with mild-moderate disability demonstrated a nearly U-shaped association between SBP and all-cause mortality, while those with severe disability exhibited a reversed J-shaped association. Specifically, SBP ranges of 135–150 mmHg for the mild-moderate disability group and 150–170 mmHg for the severe disability group were identified as reasonable blood pressure targets. Lower BP (SBP < 135mmHg, DBP < 67mmHg in the mild-moderate disability group and SBP < 150mmHg, DBP < 79mmHg in the severe disability group) was associated with an increased risk of mortality, irrespective of antihypertensive medication status. This study has taken into account competing risks and was additionally validated through sensitivity analyses. These results implied the significance of assessing disability profiles by the Barthel index in tailoring individualized BP control strategies for older individuals in long-term care setting.

### Optimal BP range for older adults with disability

The optimal BP range might be higher for disabled older adults in long-term care than for the community-dwelling older population. Previous community-based cohort studies for individuals aged between 75 and 90 years old demonstrated that SBP < 110–125 mmHg correlated with increased mortality [18, 30, 31]. In our study, SBP lower than 135 mmHg increased all-cause mortality in mild-moderate disabled participants. Furthermore, for our severe disability group, the safe SBP range was 150–170 mmHg. The optimal SBP target would change with functional status, as fibrosis of the heart and other vital organ is implicated with aging [32]. A relatively higher BP level was supposed to maintain adequate organ perfusion, such as the brain, heart, and kidneys, especially in individuals with impaired functional status and multimorbidity [33]. The benefit of antihypertensive treatment in older patients decreased with the deterioration of clinical status [34]. In addition, there is extensive documentation regarding the heightened susceptibility of older adults with functional decline to treatment-related drug side effects [35]. Importantly, our study is the first to suggest that older adults with severe disability may require a higher BP level and less aggressive antihypertensive treatment than those with mild-moderate disability. Antihypertensive treatment should be administered cautiously in this special severe disability group to avoid iatrogenic-induced problems in long-term care settings.

### Lower BP with all-cause mortality and cause-specific mortality

Our study underscores the significance of paying attention to the association between lower BP and heightened all-cause mortality in long-term care settings. Similarly, the PARTAGE study with individuals over 80 years old in institutional care showed an inverted association between increased SBP and all-cause mortality [36]. In a Swedish cohort comprising nursing home residents ≥ 65 years old, SBP < 120 mmHg was linked to elevated all-cause mortality, regardless of the use of antihypertensive medications [37]. Reduced BP is thought to be an indicator of a more advanced neurodegenerative process [38]. This explains why severe disability older adults have lower baseline BP compared to those with mild-moderate disability. It is noteworthy that the number of non-cardiovascular deaths was 2.1 times that of cardiovascular deaths in our study. This finding aligns with previous research indicating that in older adults with disabilities, the risk of non-cardiovascular mortality, particularly respiratory and infection-related deaths, may outweigh that of cardiovascular risk [39]. These results suggest that the association between lower SBP and increased risk of mortality among severely disabled older individuals is largely driven by non-cardiovascular mortality. Our data additionally indicate that higher SBP-related cardiovascular mortality have a restricted influence on overall mortality in severe disability older adults. This could be attributed to the intricate nature of co-existing comorbidities [40]. Consequently, our findings suggest that addressing non-cardiovascular morbidities may hold relatively greater significance for severe disabled older adults.

Previous research has proposed that reverse causation, wherein lower BP result from proximity to death, might contribute to the association between low BP and mortality [41]. However, another investigation demonstrated that, in a long-term care setting, SBP levels remained stable until the last 3–4 weeks of life [42]. Considering the median follow-up period in our current study, which exceeds 14.2 months, it is improbable that the observed associations are solely attributed to terminal BP decline or reverse causation. The sensitivity analysis excluding individuals who died within a 6-month period after admission further validated these findings. Future studies employing randomized interventions could help to gain a deeper understanding of this causal relationship.

### Modification effect of disability on the optimal BP range for older adults

In our study, the associations between BP and mortality risk among older adults exhibited variation according to their ADL disability status. We hypothesized that decline of biological function attenuated the correlation between elevated SBP and mortality risk. This modification effect of disability profile on the association between BP and mortality remained robust across subgroups and was independent of chronological age in our investigation. In addition, no significant interaction was found between age and SBP-mortality association, suggesting that the modification effect was mostly attributed to the ADL disability status. Biological aging process accompanied by functional dependence is more important than chorological aging in comprehensive geriatric assessments [43].

Prior studies about the association between BP and mortality in older populations using frailty measures have yielded mixed findings [44]. Some studies observed that gait speed could either alter or leave unaffected the link between SBP and mortality risk [20, 45]. Additionally, the modification effect of frailty assessed by the electronic frailty index on the association between SBP and mortality was only evident in the age group of 85 years and older [19]. The diverse measures to define frailty status across different studies may weaken the comparability of their results. Frailty is more of a reversible condition than disability [46]. Older adults who were frail but without disability had no increased risk for mortality or nursing home admission [47]. Disability may manifest later than frailty in the hierarchical continuum of the aging process and tends to be more stable over time [48]. Our study highlights that biological aging, as reflected by the degree of disability assessed through the Barthel index scores, exerts a substantial modification effect on the association between BP and mortality risk.

### Strengths and limitations

This study had several noteworthy strengths. By doubly robust analysis, we minimized bias effect in the estimation of mortality risk. Inverse propensity weighting was utilized to balance the distribution of baseline covariates across diverse BP exposure groups. Second, through data-driven methods and this large-scale long-term care cohort, we conducted a thorough exploration for the effect of disability profile on the association between BP and mortality. By the advantage of large sample size, our study provided valuable evidence for tailoring BP management in disabled older adults in the long-term care setting.

However, certain limitations should be acknowledged. Firstly, given that our participants were drawn from the LTCI, the number of severely disabled individuals considerably outweighed that of the mild-moderately disabled, potentially introducing selection bias. Secondly, data on changes in BP during follow-up were not available, preventing us from discerning the impact of BP variability on mortality. Thirdly, for some bedbound participants, the measurement of BP in recumbent position may result in slightly higher SBP and lower DBP readings compared to seated measurements [49]. Further investigations are warranted to explore the association between longitudinal BP fluctuations and mortality among older individuals.

## Conclusion

This prospective cohort study contributes further evidence regarding the increased mortality associated with low BP among older residents in long-term care settings. Furthermore, our study suggests that the disability profile modifies the associations between BP and mortality risk in older adults. A higher optimal BP range was observed in older individuals in long-term care with severe disability compared to those with mild-moderate disability. The assessment of disability using the Barthel index can serve as a valuable tool in customizing the optimal BP management strategy for long-term care older adults. The management of hypertension in long-term care residents should not be simply extrapolated from evidence obtained in older adults residing in the community.

### Supplementary Information

Supplementary Material 1.

## Data Availability

The datasets used and analyzed in this current study are available from the corresponding author on reasonable request.

## Abbreviations

BP: Blood pressure
LTCI: Long-term care insurance
ADL: Activities of daily living
SBP: Systolic blood pressure
DBP: Diastolic blood pressure
