# Supplementary material for: Disability level’s impact on blood pressure-mortality association in older long-term care adults: evidence from a large Chinese cohort study
Source: BMC Geriatr. 2024 May 31;24:478. doi: 10.1186/s12877-024-05094-y (PMC11143654; doi:10.1186/s12877-024-05094-y)
Supplement: Supplementary file 1 — Supplementary Material 1. [file 12877_2024_5094_MOESM1_ESM.docx]

|  | No. of Participants | Mild-moderate disability | |  | Severe disability | |
| --- | --- | --- | --- | --- | --- | --- |
| Blood pressure values |  | Unadjusted HR  (95% CI) | Adjusted HR  (95% CI) |  | Unadjusted HR  (95% CI) | Adjusted HR  (95% CI) |
| **Systolic blood pressure (mmHg)** | |  |  |  |  |  |
| <120 | 9,717 | **1.48 (1.30, 1.67)** | **1.36 (1.19, 1.55)** |  | **1.49 (1.41, 1.57)** | **1.39 (1.32, 1.47)** |
| 120–129 | 6,423 | 1.13 (0.98, 1.29) | 1.12 (0.98, 1.29) |  | **1.24 (1.17, 1.32)** | **1.19 (1.12, 1.26)** |
| 130–139 | 6,958 | 0.94 (0.83, 1.08) | 0.96 (0.84, 1.10) |  | **1.10 (1.04, 1.17)** | **1.06 (1.00, 1.12)** |
| 140–149 (reference) | 5,953 | 1.00 | 1.00 |  | 1.00 | 1.00 |
| 150–159 | 4,417 | 0.97 (0.84, 1.11) | 1.03 (0.90, 1.18) |  | 0.97 (0.91, 1.04) | 0.99 (0.92, 1.06) |
| 160–169 | 3,282 | 0.90 (0.77, 1.04) | 0.90 (0.76, 1.05) |  | 0.92 (0.85, 0.99) | 0.95 (0.88, 1.03) |
| 170–179 | 1,967 | 0.84 (0.71, 1.00) | 0.86 (0.72, 1.03) |  | 0.87 (0.79, 0.96) | **0.87 (0.78, 0.96)** |
| 180–189 | 1,187 | 0.93 (0.76, 1.14) | 1.14 (0.91, 1.42) |  | 0.92 (0.82, 1.03) | 0.98 (0.86, 1.12) |
| ≥190 | 1,100 | 0.90 (0.72, 1.11) | 0.94 (0.73, 1.21) |  | 0.85 (0.75, 0.95) | 0.93 (0.82, 1.06) |
| **Diastolic blood pressure (mmHg)** | |  |  |  |  |  |
| <70 | 13,439 | **1.45 (1.31, 1.60)** | **1.22 (1.10, 1.36)** |  | **1.39 (1.33, 1.46)** | **1.24 (1.18, 1.29)** |
| 70–79 | 10,807 | 1.08 (0.97, 1.20) | 1.03 (0.92, 1.14) |  | **1.07 (1.02, 1.12)** | 1.03 (0.98, 1.08) |
| 80–89 (reference) | 9,356 | 1.00 | 1.00 |  | 1.00 | 1.00 |
| ≥90 | 7,402 | **0.87 (0.78, 0.98)** | 0.98 (0.87, 1.11) |  | **0.87 (0.83, 0.92)** | 0.98 (0.93, 1.04) |

**Table S1.** Association of blood pressure as categorical variable with all-cause mortality risk stratified by disability status.

Hazard Ratios were shown in bold if with significant difference.

**Table S2.** Association of blood pressure range with cardiovascular and non-cardiovascular mortality risk stratified by disability status.

|  | No. of  Participants | No. of  events | Cardiovascular mortality | | No. of  events | Non-Cardiovascular mortality | |
| --- | --- | --- | --- | --- | --- | --- | --- |
| Blood pressure values |  |  | Unadjusted HR  (95% CI) | Adjusted HR  (95% CI) |  | Unadjusted HR  (95% CI) | Adjusted HR  (95% CI) |
| ***Mild-moderate disability group*** |  |  |  |  |  |  |  |
| **Systolic blood pressure, mmHg** |  |  |  |  |  |  |  |
| Lower (<135 mmHg) | 3,077 | 282 | 0.98 (0.82, 1.17) | 0.98 (0.82, 1.17) | 877 | **1.43 (1.28, 1.60)** | **1.31 (1.17, 1.47)** |
| Middle (135-150mmHg) | 2,131 | 227 | 1.00 | 1.00 | 481 | 1.00 | 1.00 |
| Higher (＞150 mmHg) | 2,927 | 347 | 1.05 (0.89, 1.24) | 1.10 (0.93, 1.30) | 616 | 0.88 (0.78, 0.99) | 0.91 (0.81, 1.03) |
| **Diastolic blood pressure, mmHg** |  |  |  |  |  |  |  |
| Lower (< 67 mmHg) | 1,588 | 179 | 1.16 (0.98, 1.38) | 1.02 (0.85, 1.23) | 549 | **1.61 (1.46, 1.79)** | **1.41 (1.27, 1.57)** |
| Middle (67-90mmHg) | 4,964 | 515 | 1.00 | 1.00 | 1,136 | 1.00 | 1.00 |
| Higher (＞90 mmHg) | 1,583 | 162 | 0.96 (0.80, 1.15) | 1.10 (0.91, 1.33) | 289 | 0.78 (0.68, 0.88) | 0.95 (0.83, 1.09) |
| ***Severe disability group*** |  |  |  |  |  |  |  |
| **Systolic blood pressure, mmHg** |  |  |  |  |  |  |  |
| Lower (<150 mmHg) | 24,029 | 3,603 | **1.19 (1.10, 1.28)** | **1.14 (1.06, 1.24)** | 7,808 | 1.16 (0.98, 1.38) | 1.02 (0.85, 1.23) |
| Middle (150-170mmHg) | 6,070 | 862 | 1.00 | 1.00 | 1,625 | 1.00 | 1.00 |
| Higher (＞170mmHg) | 2,770 | 418 | 1.04 (0.92, 1.16) | 1.07 (0.94, 1.22) | 651 | 0.96 (0.8, 1.15) | 1.10 (0.91, 1.33) |
| **Diastolic blood pressure, mmHg** |  |  |  |  |  |  |  |
| Lower (<79 mmHg) | 19,345 | 3,032 | **1.21 (1.13, 1.29)** | **1.11 (1.04, 1.19)** | 6,539 | **1.30 (1.24, 1.36)** | **1.17 (1.11, 1.23)** |
| Middle (79 - 90mmHg) | 8,771 | 1,212 | 1.00 | 1.00 | 2,441 | 1.00 | 1.00 |
| Higher (＞90 mmHg) | 4,753 | 639 | 0.97 (0.88, 1.07) | 1.06 (0.95, 1.17) | 1,104 | 0.83 (0.78, 0.89) | 0.97 (0.90, 1.05) |

Hazard Ratios were shown in bold if with significant difference.

**Table S3.** Association of blood pressure as categorical variable with cardiovascular mortality risk stratified by disability status

|  | No. of  Participants | No. of  events | Mild-moderate disability | | No. of  deaths | Severe disability | |
| --- | --- | --- | --- | --- | --- | --- | --- |
| Blood pressure values |  |  | Unadjusted HR  (95% CI) | Adjusted HR  (95% CI) |  | Unadjusted HR  (95% CI) | Adjusted HR  (95% CI) |
| **Systolic blood pressure (mmHg)** |  |  |  |  |  |  |  |
| <120 | 9,717 | 126 | 1.02 (0.80, 1.29) | 1.06 (0.82, 1.36) | 1,332 | **1.38 (1.26, 1.52)** | **1.33 (1.21, 1.46)** |
| 120–129 | 6,423 | 105 | 1.00 (0.77, 1.28) | 1.03 (0.80, 1.33) | 763 | **1.13 (1.02, 1.25)** | 1.09 (0.98, 1.21) |
| 130–139 | 6,958 | 105 | 0.74 (0.57, 0.95) | 0.77 (0.60, 1.00) | 838 | **1.11 (1.00, 1.23)** | 1.07 (0.97, 1.19) |
| 140–149 (reference) | 5,953 | 145 | 1.00 | 1.00 | 670 | 1.00 | 1.00 |
| 150–159 | 4,417 | 124 | 0.98 (0.77, 1.25) | 1.06 (0.83, 1.36) | 466 | 0.97 (0.86, 1.09) | 0.97 (0.86, 1.09) |
| 160–169 | 3,282 | 108 | 1.12 (0.87, 1.44) | 1.16 (0.90, 1.51) | 355 | 1.02 (0.90, 1.16) | 1.03 (0.90, 1.18) |
| 170–179 | 1,967 | 64 | 0.95 (0.70, 1.27) | 0.97 (0.72, 1.32) | 210 | 1.00 (0.85, 1.16) | 0.94 (0.80, 1.12) |
| 180–189 | 1,187 | 41 | 0.98 (0.69, 1.40) | 1.20 (0.83, 1.73) | 131 | 1.09 (0.90, 1.31) | 1.15 (0.94, 1.41) |
| ≥190 | 1,100 | 38 | 1.07 (0.75, 1.53) | 0.97 (0.65, 1.45) | 118 | 1.01 (0.83, 1.23) | 1.10 (0.89, 1.36) |
| **Diastolic blood pressure (mmHg)** | |  |  |  |  |  |  |
| <70 | 1,3439 | 239 | 1.10 (0.92, 1.33) | 0.94 (0.78, 1.14) | 1,879 | **1.29 (1.20, 1.39)** | **1.17 (1.08, 1.26)** |
| 70–79 | 1,0807 | 202 | 0.88 (0.73, 1.07) | 0.84 (0.69, 1.01) | 1,236 | 1.01 (0.93, 1.10) | 0.97 (0.90, 1.06) |
| 80–89 (reference) | 9,356 | 220 | 1.00 | 1.00 | 1,025 | 1.00 | 1.00 |
| ≥90 | 7,402 | 195 | 0.95 (0.78, 1.15) | 1.06 (0.87, 1.30) | 743 | 0.94 (0.85, 1.03) | 1.01 (0.92, 1.12) |

Hazard Ratios were shown in bold if with significant difference.

**Table S4.** Association of blood pressure as categorical variable with non-cardiovascular mortality risk stratified by disability status

|  | No. of  Participants | No. of  events | Mild-moderate disability | | No. of  deaths | Severe disability | |
| --- | --- | --- | --- | --- | --- | --- | --- |
| Blood pressure values |  |  | Unadjusted HR  (95% CI) | Adjusted HR  (95% CI) |  | Unadjusted HR  (95% CI) | Adjusted HR  (95% CI) |
| **Systolic blood pressure (mmHg)** | |  |  |  |  |  |  |
| <120 | 9,717 | 440 | **1.70 (1.46, 1.97)** | **1.50 (1.29, 1.75)** | 2,999 | **1.54 (1.45, 1.64)** | **1.42 (1.33, 1.52)** |
| 120-129 | 6,423 | 263 | **1.19 (1.01, 1.40)** | 1.17 (0.99, 1.38) | 1,774 | **1.30 (1.21, 1.40)** | **1.23 (1.15, 1.33)** |
| 130-139 | 6,958 | 310 | 1.04 (0.89, 1.22) | 1.05 (0.89, 1.23) | 1,685 | **1.10 (1.03, 1.18)** | 1.05 (0.98, 1.13) |
| 140-149 (reference) | 5,953 | 302 | 1.00 | 1.00 | 1,350 | 1.00 | 1.00 |
| 150-159 | 4,417 | 251 | 0.96 (0.81, 1.13) | 1.02 (0.86, 1.20) | 941 | 0.97 (0.89, 1.05) | 1.00 (0.92, 1.09) |
| 160-169 | 3,282 | 158 | **0.79 (0.65, 0.95)** | **0.78 (0.64, 0.95)** | 609 | 0.87 (0.79, 0.96) | 0.91 (0.83, 1.01) |
| 170-179 | 1,967 | 111 | **0.79 (0.64, 0.98)** | 0.81 (0.64, 1.02) | 344 | **0.81 (0.72, 0.91)** | **0.83 (0.72, 0.95)** |
| 180-189 | 1,187 | 79 | 0.91 (0.71, 1.17) | 1.11 (0.84, 1.46) | 202 | 0.83 (0.72, 0.96) | 0.90 (0.76, 1.06) |
| ≥190 | 1,100 | 60 | 0.81 (0.61, 1.07) | 0.94 (0.69, 1.29) | 180 | **0.76 (0.65, 0.89)** | 0.84 (0.71, 1.00) |
| **Diastolic blood pressure (mmHg)** | |  |  |  |  |  |  |
| <70 | 1,3439 | 686 | **1.62 (1.44, 1.83)** | **1.36 (1.20, 1.54)** | 4,105 | **1.45 (1.37, 1.53)** | **1.27 (1.20, 1.34)** |
| 70–79 | 1,0807 | 526 | **1.18 (1.04, 1.34)** | 1.12 (0.98, 1.28) | 2,645 | **1.10 (1.04, 1.17)** | 1.05 (0.99, 1.12) |
| 80–89 (reference) | 9,356 | 429 | 1.00 | 1.00 | 2,013 | 1.00 | 1.00 |
| ≥90 | 7,402 | 333 | 0.83 (0.72, 0.96) | 0.95 (0.81, 1.10) | 1,321 | **0.84 (0.79, 0.90)** | 0.97 (0.90, 1.04) |

Hazard Ratios were shown in bold if with significant difference.


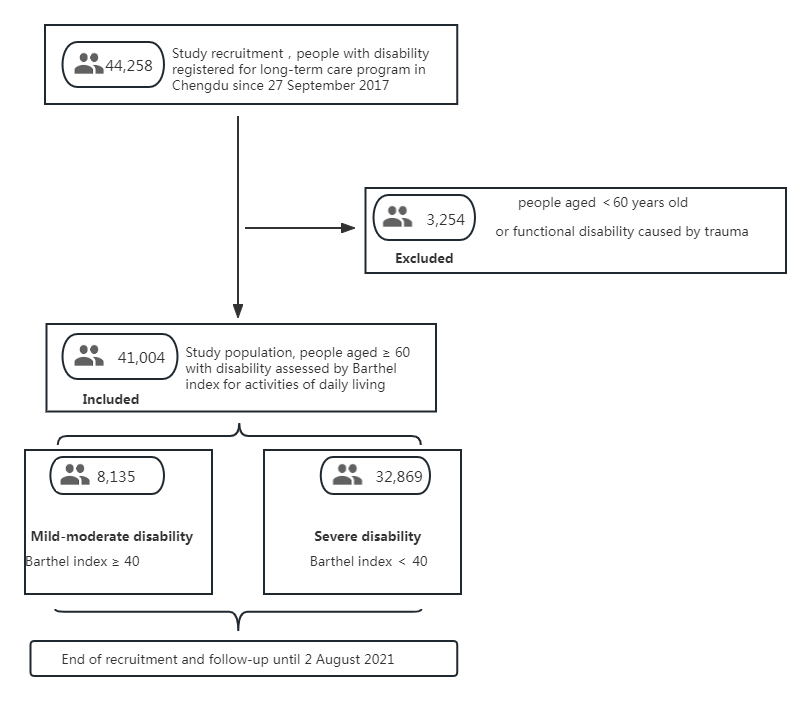


**Figure S1**. Flowchart of the inclusion of participants.


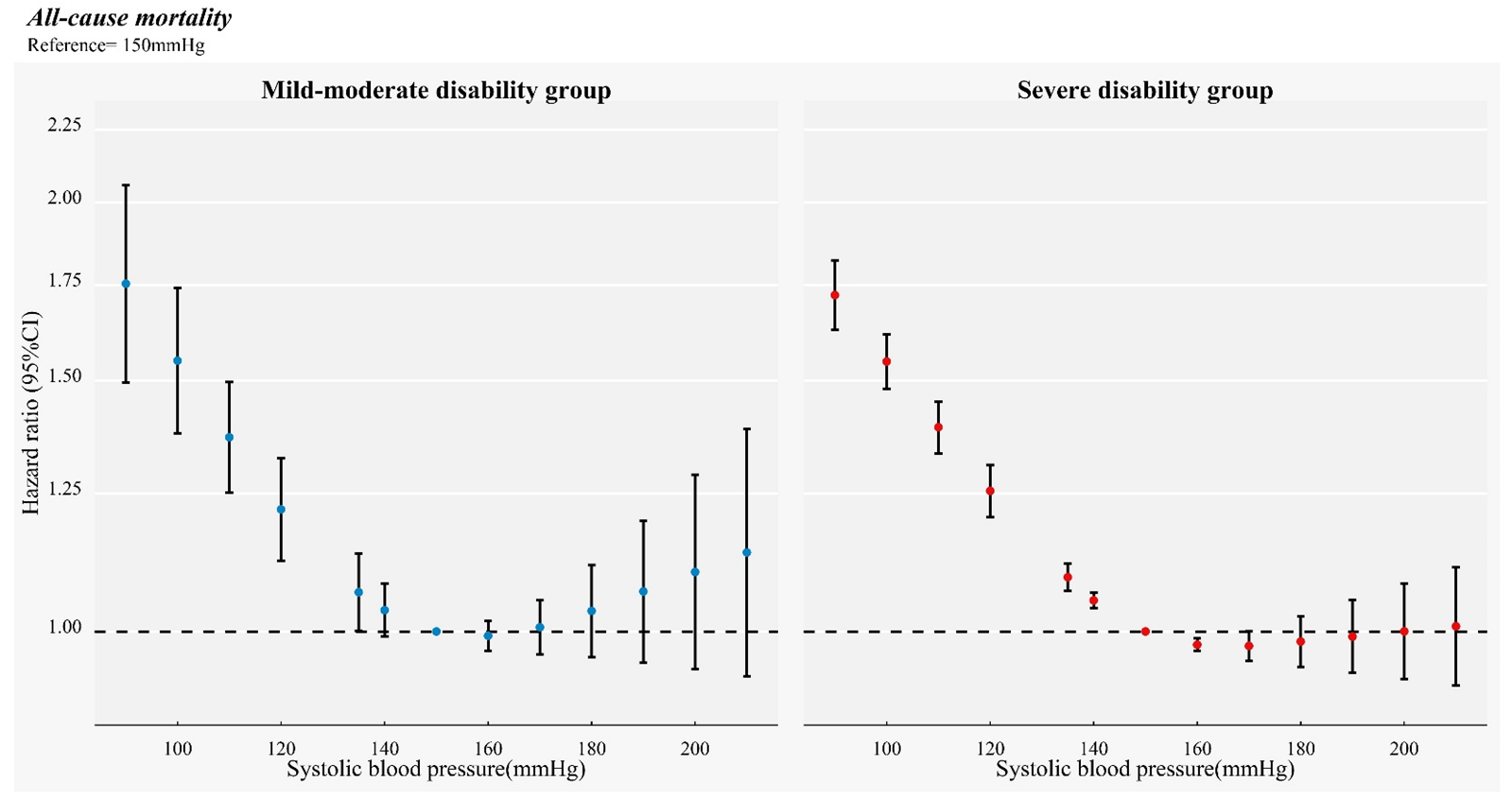


**Figure S2.** Hazard ratio and 95% confidence intervals for all-cause mortality at different levels of systolic blood pressure in Cox models with restricted cubic splines after adjustment (150 mm Hg reference).

**
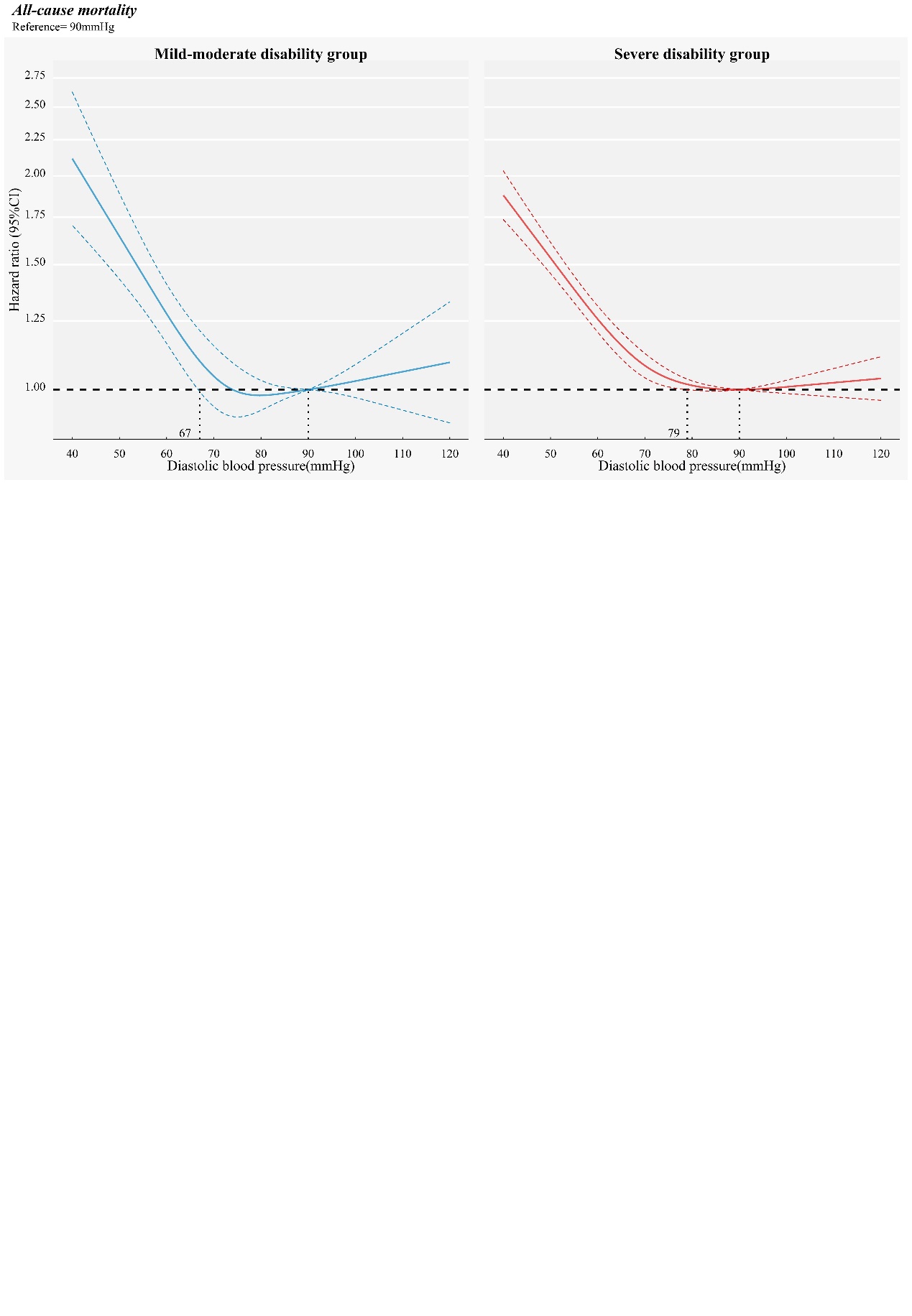
**

**Figure S3.** Cox models with cubic restricted splines of the association between diastolic blood pressure and all-cause mortality risk stratified by disability status (reference: 90 mmHg). Reversed J-shaped association was both shown in the mild-moderate and severe disability groups. Hazard ratio was adjusted for age, sex, education, marital status, care modes, multimorbidity, cognitive impairment and perception impairment.


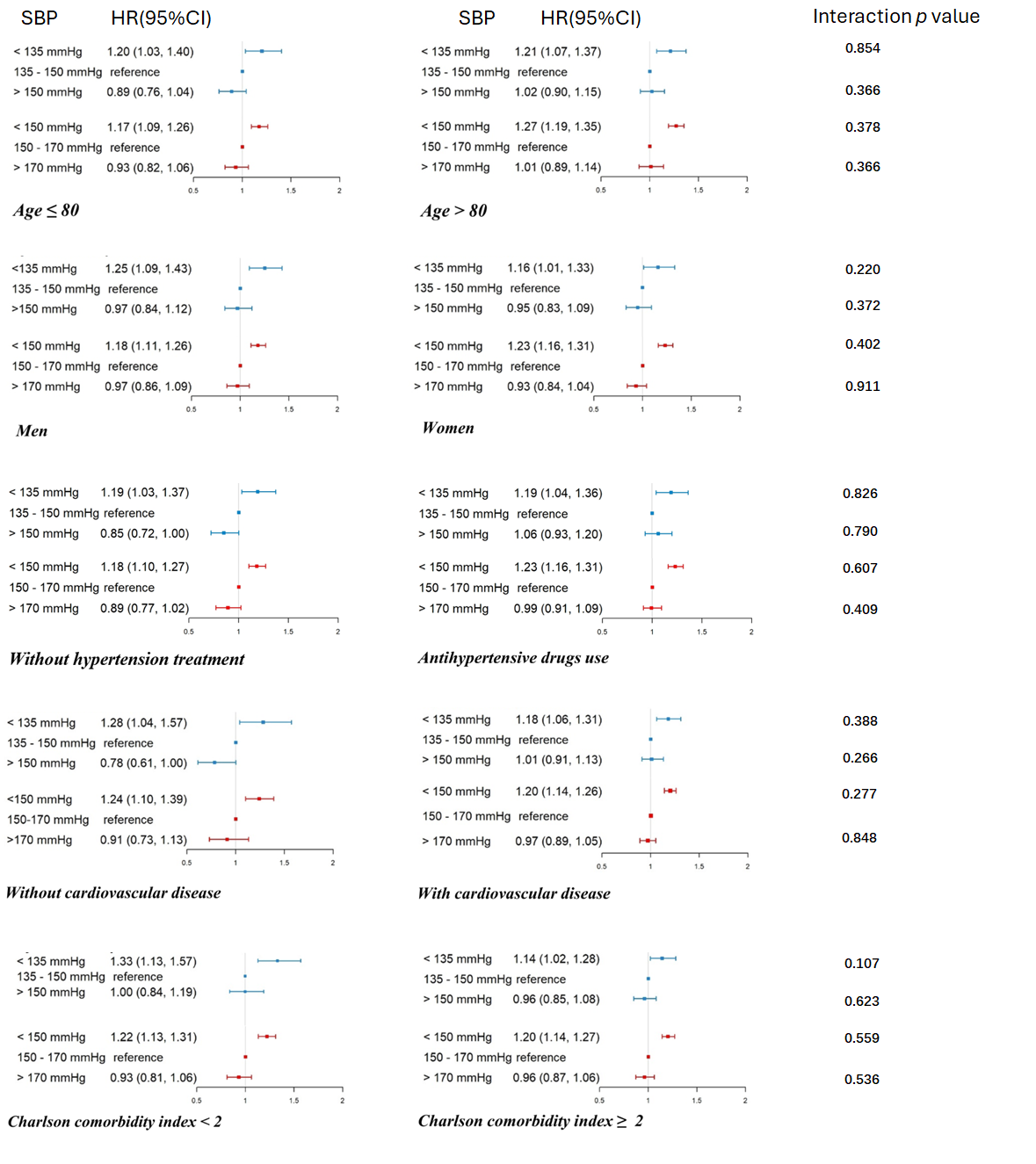


**Figure S4.** Hazard ratio for all-cause mortality associated with systolic blood pressure by subgroups. HRs (95% CI) were adjusted for age, sex, education, marital status, care modes, multimorbidity, cognitive impairment and perception impairment. Blue lines for mild-moderate disability group, red lines for severe disability group.

**
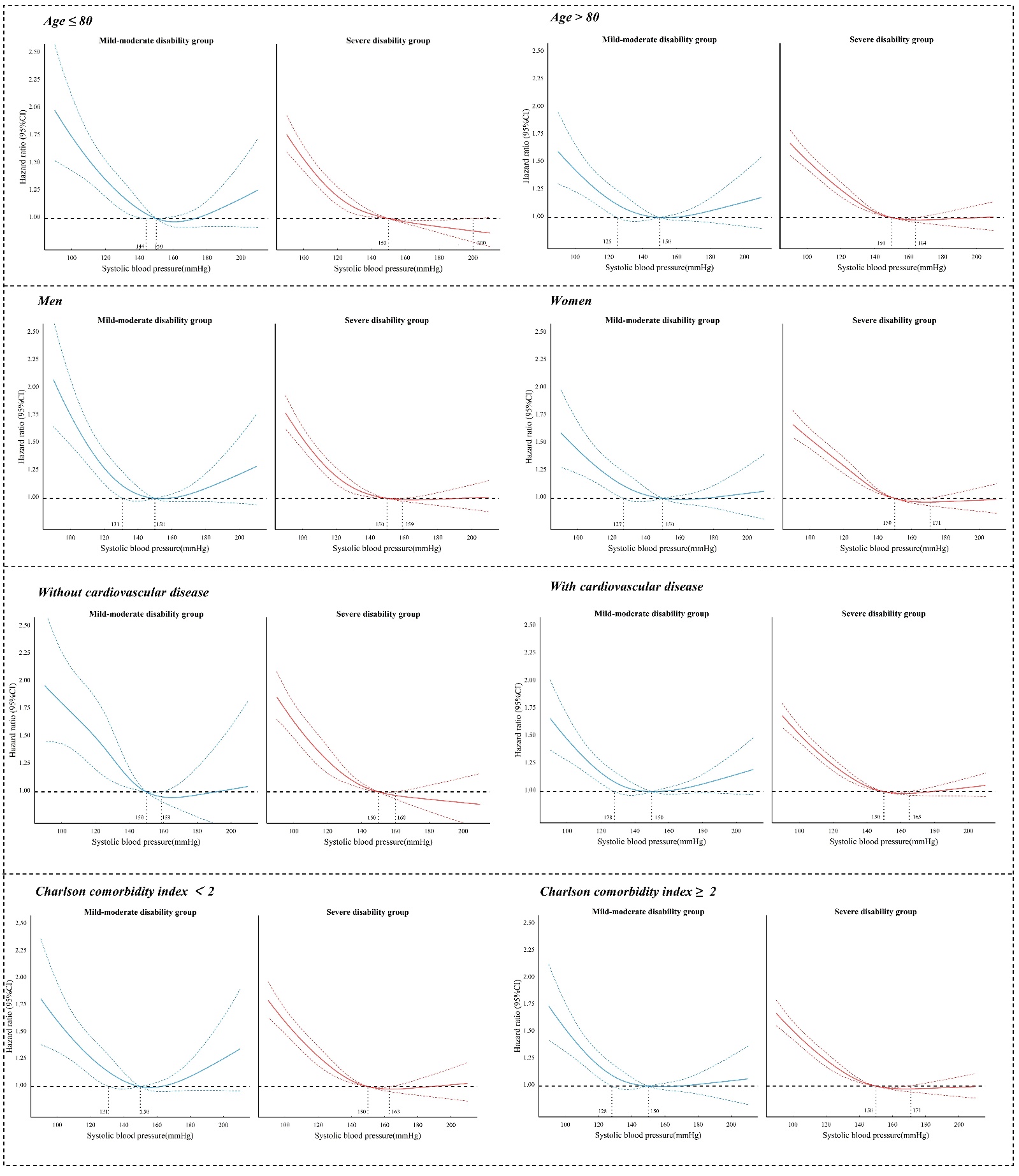
**

**Figure S5.** Non-linear trend for all-cause mortality associated with systolic blood pressure by subgroups.


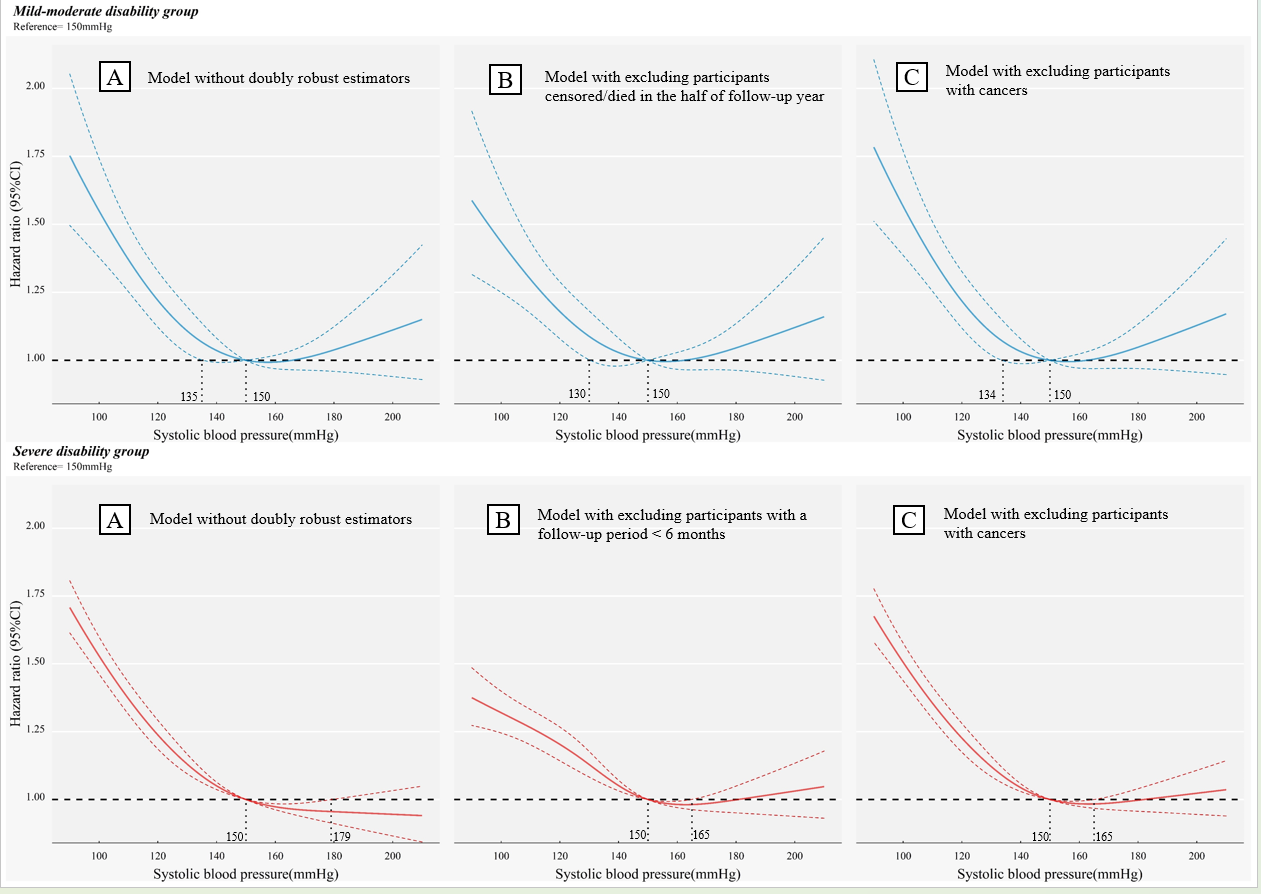


**Figure S6**. Sensitivity analysis of the association between SBP and all-cause mortality stratified by disability status. A: The full models were fitted for Hazard ratio of mortality with Cox model by cubic restricted splines and adjusted for age, sex, education, marital status, care modes, multimorbidity, cognitive impairment and perception impairment. B: The full models were excluding participants with a follow-up period < 6 months. C: The full models were excluding participants with cancers.
